# Supplementary material for: Elevated growth and biomass along temperate forest edges
Source: Nat Commun. 2021 Dec 10;12:7181. doi: 10.1038/s41467-021-27373-7 (PMC8664805; doi:10.1038/s41467-021-27373-7)
Supplement: Supplementary file 1 — Supplementary Information [file 41467_2021_27373_MOESM1_ESM.pdf]

Supporting information for *Elevated growth and biomass along temperate forest edges*

Luca L. Morreale[1, 2,*], Jonathan R. Thompson[2], Xiaojing Tang[1], Andrew B. Reinmann[3,4,5], Lucy R. Hutyra[1].

[1] Department of Earth & Environment, Boston University

[2] Harvard Forest, Harvard University

[3] Environmental Science Initiative, CUNY Advanced Science Research Center

[4] Graduate Program in Earth and Environmental Sciences and Biology, CUNY Graduate Center

[5] Department of Geography and Environmental Sciences, Hunter College

* Corresponding Author; *lmorreal@bu.edu*

Supplementary Figures

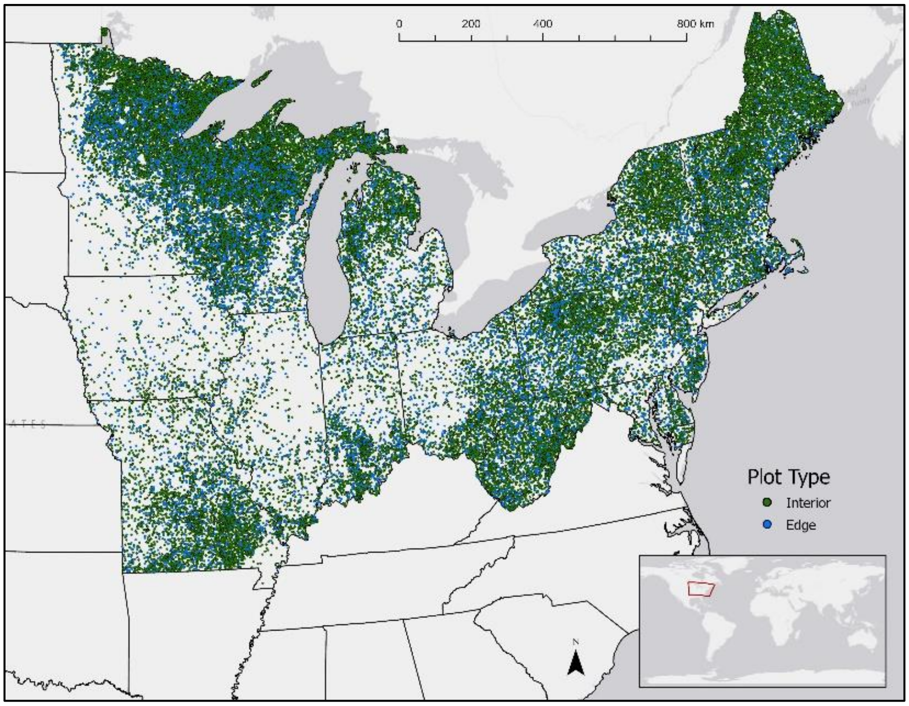

Supplementary Figure 1. Study region and approximate locations of edge and interior FIA plots.

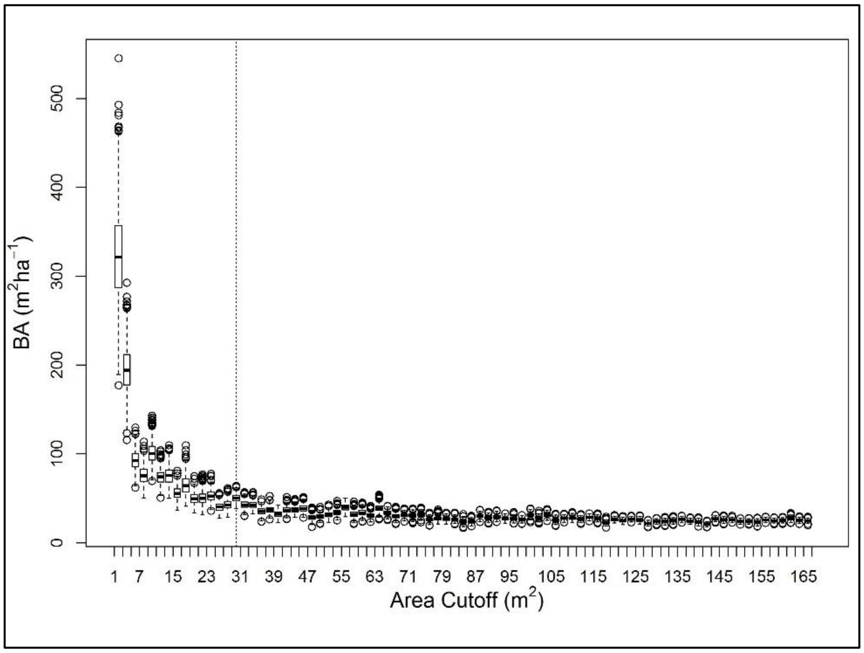

Supplementary Figure 2. Sensitivity of basal area estimates to subplot area. FIA plots with an area of <30m^2 (vertical line) were excluded from this analysis due to small area biases. n = 10583 forest subplots were used for this sensitivity analysis. Boxplots indicate median (center line), 25th and 75th percentile (box) 5th and 95th percentile (whiskers), and outliers (individual points).

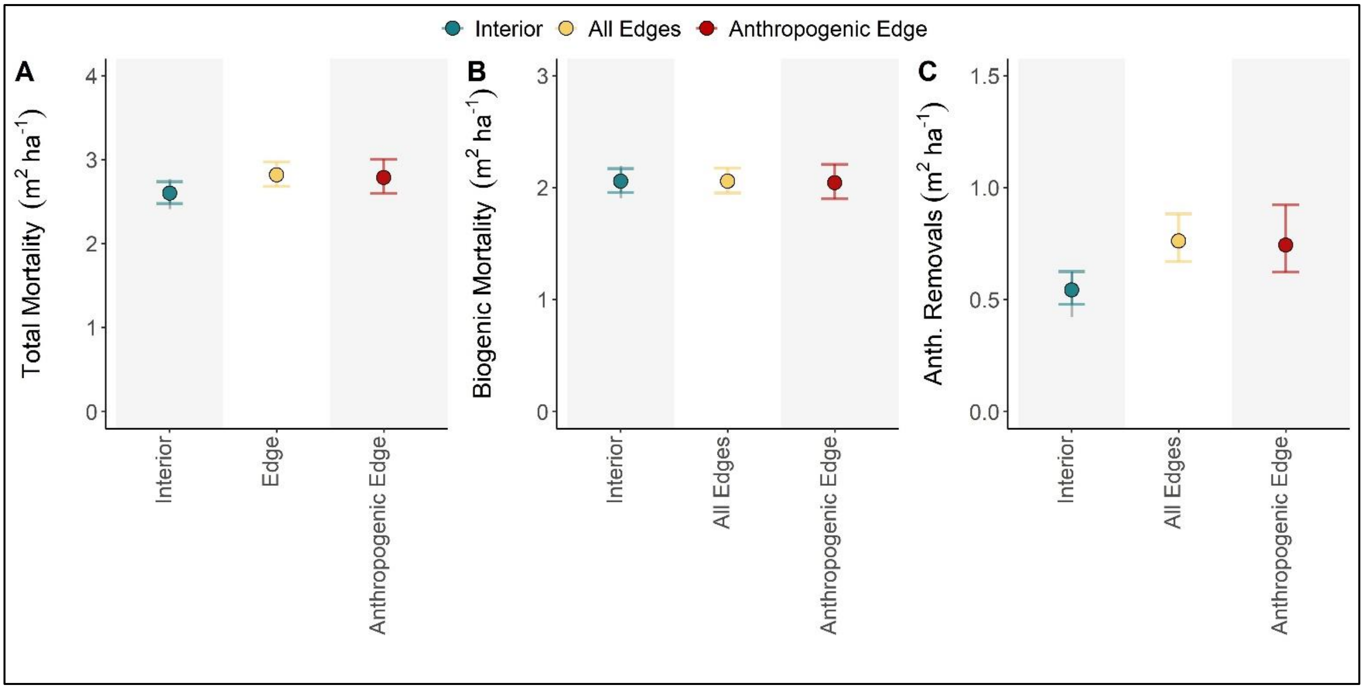

Supplementary Figure 3. Mortality differences between edge, interior, and anthropogenic edges. (A) Combined biogenic mortality and anthropogenic removals. (B) Mortality from biogenic causes, measured in BA of dead trees that remained on the subplot. (C) Mortality from anthropogenic cutting, measured in BA of trees that were cut and removed from the subplot. Error bars show 95% confidence intervals on mean marginal effects. Interior and All Edge groups have n = 6607 independent subplots, Anthropogenic Edges have n = 4327 independent subplots

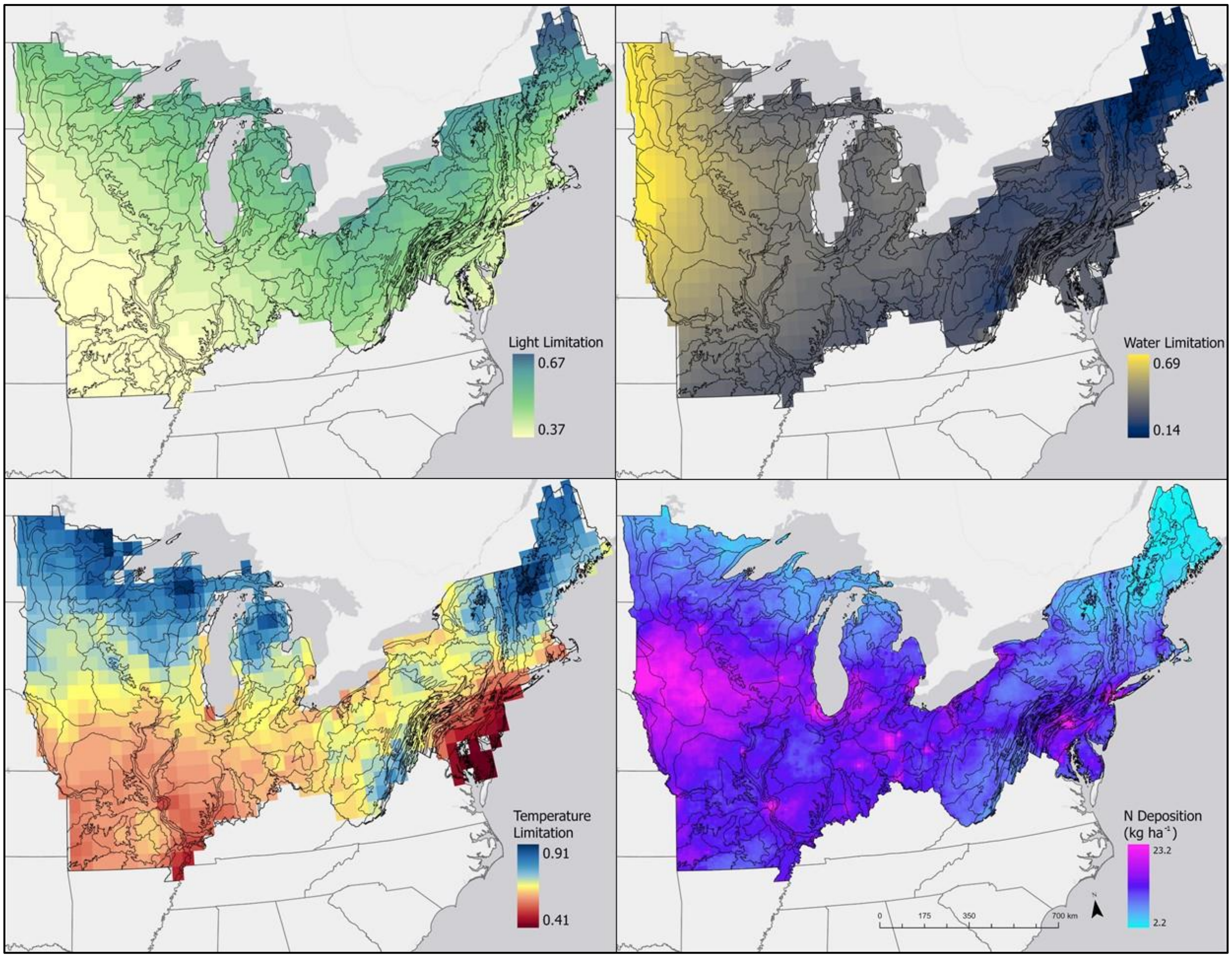

Supplementary Figure 4. Gridded abiotic predictors of forest productivity. (A) Light limitation on vegetation productivity, unit-index ranging from 0 to 1 [6]. (B) Water limitation on vegetation productivity, unit-index ranging from 0 to 1 [6]. (C) Temperature limitation on vegetation productivity, unit-index ranging from 0 to 1 [6]. (D) Atmospheric nitrogen deposition in 2018, kg ha^{-1} [7].

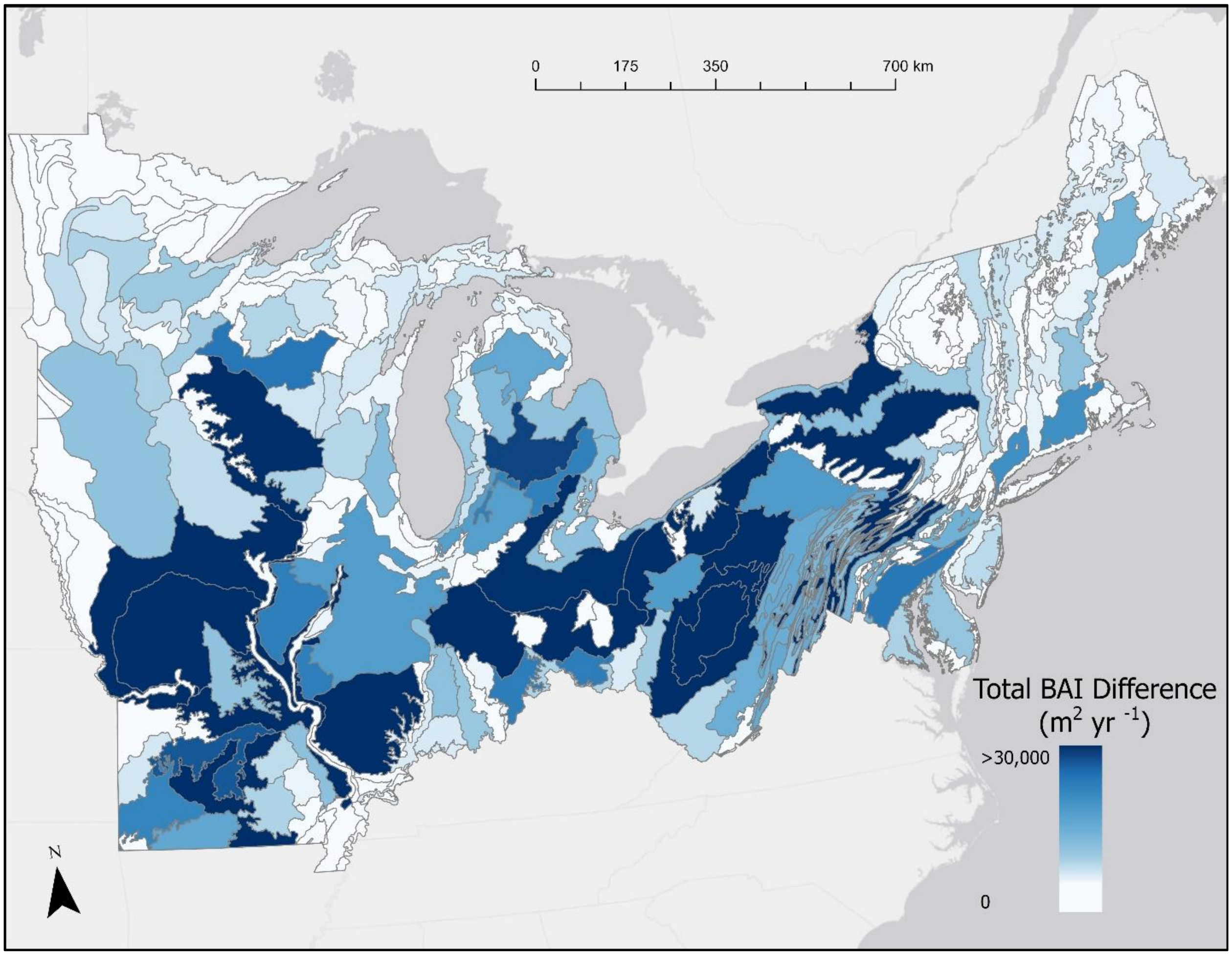

Supplementary Figure 5. Increases in total ecoregion BAI (m^2 yr^{-1}) associated with elevated growth at the forest edge. BAI difference was calculated from individual ecoregion forest composition and forest edge area.

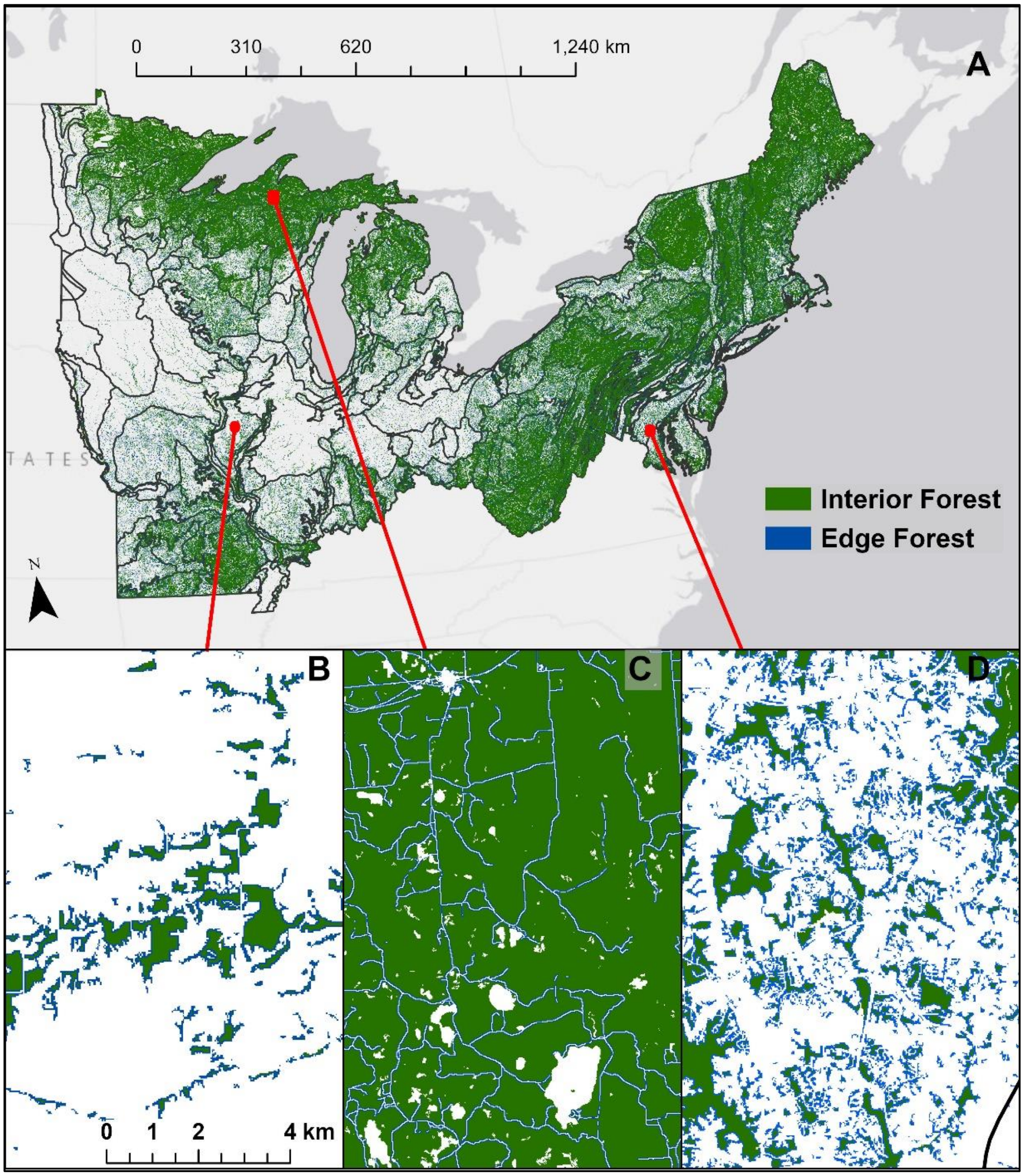

Supplementary Figure 6. (A) Edge and interior forest cover designated from the 2016 National Land Cover Database [25]. (B) Agricultural areas have a high proportion of edge forest (Illinois). (C) The northern areas in our study region have the smallest amount of fragmentation (Michigan). (D) The metropolitan east coast is both heavily fragmented and moderately forested (Maryland). EPA Level IV ecoregion boundaries are shown in black.

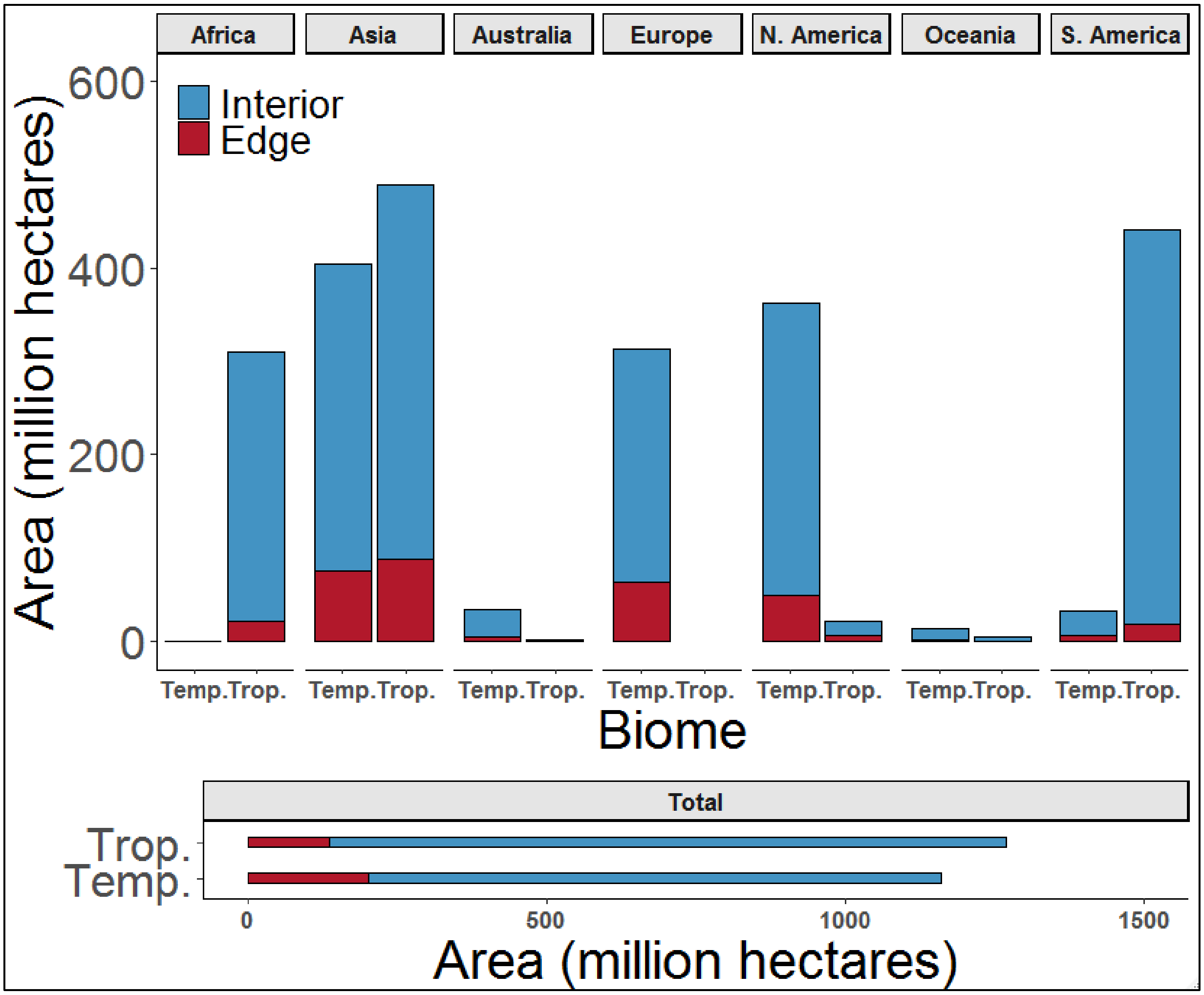

Supplementary Figure 7. Results from the robustness test of % forest cover threshold for estimates of temperate and tropical fragmentation using a 30% forest cover minimum definition of forest.

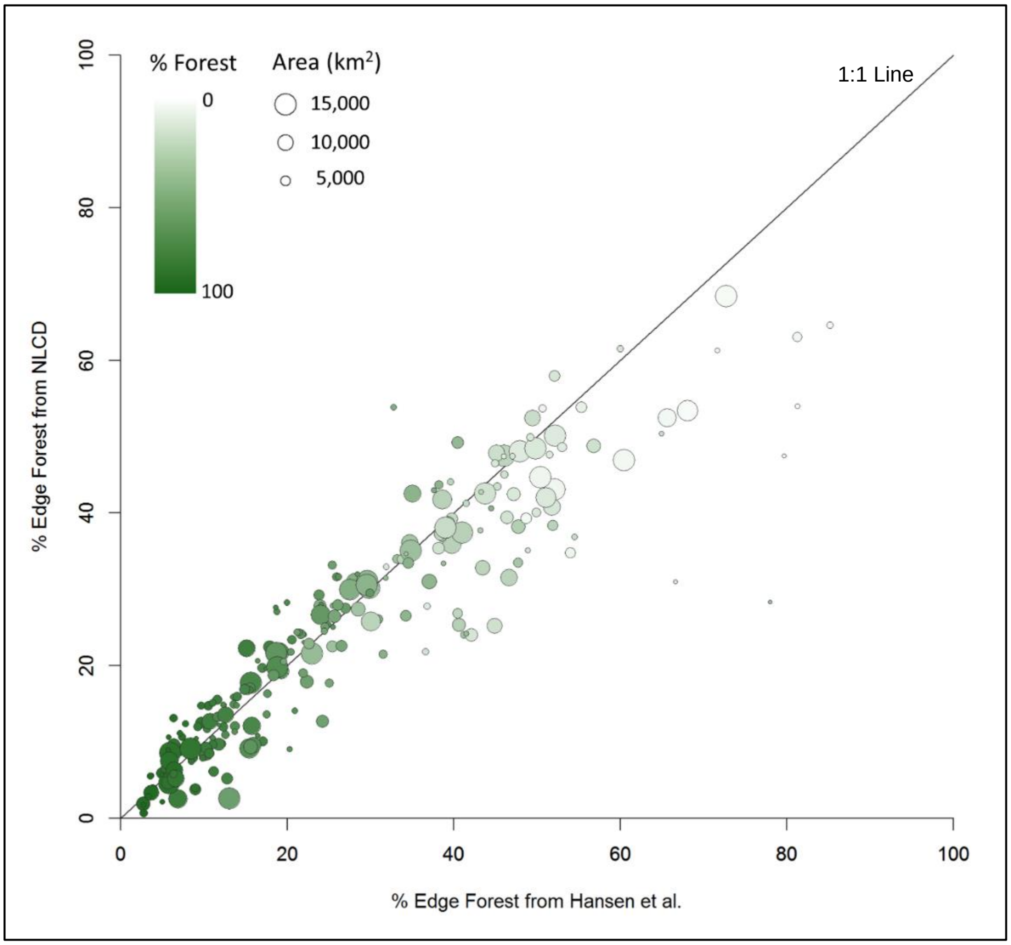

Supplementary Figure 8. Percent edge forest of 247 ecoregions located in the Northeast US derived from Hansen Global Forest Change (v1.7) 16 dataset versus from NLCD land cover map 13. Size of the points corresponds to the size of the ecoregions, color of the points corresponds to percent forest cover within each ecoregion.

Supplementary Tables

Supplementary Table 1. Definition of forest type groups with sample size used in final analyses.

Forest Type Name	FIA Forest Type Codes	N - All Edges	N -Anthropogenic Edges
Northern Pines - Hemlock	100 - 105	491	247
Spruce - Fir	120 - 129	402	98
Southern Conifers	140 - 391	123	75
Oak - Pine	400 - 409	248	156
Oak - Hickory	500 - 520	2551	1831
Bottomland Forests	600 - 709	693	329
Northern Hardwood	800 - 809	1229	773
Aspen - Birch	900 - 905	776	305

Supplementary Table 2. Model forms and fits from GLMs with BA and BAI as response variables. dAIC is the difference in AIC relative to the best model within each model set. Pseudo R^2 = Nagelkerke pseudo R^2, a goodness of fit metric calculated from model likelihood. Resid. Dev = Residual deviance, a generalization of residual sum of squares. Resid. Df = Residual degrees of freedom used in the calculation of residual deviance.

	Response Var.	Model Form	Pseudo R^2	Resid. Dev	Resid. DF	AIC	dAIC
All Edges							
	**BA**	**EdgeType * ForestType + Light + Water + Temperature + N.Dep**	**0.069**	**6971.6**	**13652**	**109273.2**	**0.0**
	BA	EdgeType * ForestType + Water	0.067	6986.6	13655	109299.0	25.7
	BA	EdgeType * ForestType + Temperature	0.060	7034.7	13655	109400.8	127.5
	BA	EdgeType * ForestType + Light	0.057	7054.0	13655	109441.2	167.9
	BA	EdgeType + ForestType	0.055	7065.9	13664	109448.3	175.1
	BA	EdgeType * ForestType + N.Dep	0.056	7060.5	13655	109454.9	181.7
	BA	EdgeType * ForestType	0.056	7062.0	13656	109456.0	182.8
	BA	ForestType	0.047	7123.3	13665	109566.2	293.0
	BA	EdgeType	0.008	7386.7	13672	110091.4	818.2
Anthro. Edges							
	**BA**	**EdgeType * ForestType + Light + Water + Temperature + N.Dep**	**0.059**	**4015.5**	**8088**	**65637.3**	**0.0**
	BA	EdgeType * ForestType + Water	0.058	4022.8	8091	65647.2	10.0
	BA	EdgeType * ForestType + Temperature	0.053	4041.7	8091	65688.3	51.1
	BA	EdgeType * ForestType + Light	0.051	4046.8	8091	65699.4	62.1
	BA	EdgeType + ForestType	0.049	4056.0	8100	65701.2	64.0
	BA	EdgeType * ForestType + N.Dep	0.051	4049.8	8091	65705.9	68.6

	BA	EdgeType * ForestType	0.050	4051.2	8092	65706.8	69.6
	BA	ForestType	0.031	4128.3	8101	65854.4	217.2
	BA	EdgeType	0.019	4175.1	8108	65939.3	302.1
All Edges							
	**BAI**	**EdgeType * ForestType + Light + Water + Temperature + N.Dep**	**0.153**	**6977.2**	**13652**	**7718.4**	**0.0**
	BAI	EdgeType * ForestType + Temperature	0.122	7075.9	13655	7920.5	202.1
	BAI	EdgeType * ForestType + Water	0.121	7082.0	13655	7933.1	214.7
	BAI	EdgeType * ForestType + N.Dep	0.118	7091.1	13655	7952.3	234.0
	BAI	EdgeType * ForestType + Light	0.107	7126.9	13655	8027.0	308.6
	BAI	EdgeType * ForestType	0.106	7129.9	13656	8031.1	312.7
	BAI	EdgeType + ForestType	0.102	7142.4	13664	8041.2	322.8
	BAI	ForestType	0.053	7301.4	13665	8366.1	647.7
	BAI	EdgeType	0.049	7313.1	13672	8375.9	657.6
Anthro. Edges							
	**BAI**	**EdgeType * ForestType + Light + Water + Temperature + N.Dep**	**0.149**	**3792.7**	**8088**	**5409.1**	**0.0**
	BAI	EdgeType * ForestType + N.Dep	0.131	3827.5	8091	5483.0	74.0
	BAI	EdgeType * ForestType + Temperature	0.130	3830.6	8091	5490.0	80.9
	BAI	EdgeType * ForestType + Water	0.128	3833.4	8091	5496.4	87.3
	BAI	EdgeType * ForestType + Light	0.126	3838.0	8091	5506.9	97.8
	BAI	EdgeType * ForestType	0.124	3841.7	8092	5513.2	104.1
	BAI	EdgeType + ForestType	0.118	3853.5	8100	5524.1	115.0
	BAI	EdgeType	0.098	3894.8	8108	5601.2	192.2
	BAI	ForestType	0.022	4046.1	8101	5949.1	540.0

Supplementary Table 3. Predicted mean marginal effects for each forest type and edge group. Upper and lower 95% confidence intervals in parentheses. Marginal effects were calculated with other predictors held at their within-forest type means. All reported values are in the units of BAI and BA (m^2 ha $^{-1}$ yr $^{-1}$ and m^2 ha $^{-1}$, respectively).

Unit	Forest Type	Edge Type	Predicted
BAI	All Forests	Interior	0.51 (0.5-0.51)
BAI	All Forests	Edge	0.63 (0.62-0.64)
BAI	All Forests	Anthropogenic Edge	0.71 (0.7-0.72)
BAI	Aspen - Birch	All Edges	0.5 (0.48-0.53)

BAI	Aspen - Birch	Interior	0.46 (0.44-0.48)
BAI	Aspen - Birch	Anthropogenic Edge	0.62 (0.58-0.66)
BAI	Bottomland Forests	All Edges	0.62 (0.59-0.65)
BAI	Bottomland Forests	Interior	0.52 (0.49-0.54)
BAI	Bottomland Forests	Anthropogenic Edge	0.7 (0.66-0.75)
BAI	Northern Hardwood	All Edges	0.64 (0.61-0.66)
BAI	Northern Hardwood	Interior	0.52 (0.5-0.53)
BAI	Northern Hardwood	Anthropogenic Edge	0.69 (0.66-0.72)
BAI	Northern Pines - Hemlock	All Edges	0.72 (0.68-0.76)
BAI	Northern Pines - Hemlock	Interior	0.62 (0.59-0.65)
BAI	Northern Pines - Hemlock	Anthropogenic Edge	0.78 (0.73-0.84)
BAI	Oak - Hickory	All Edges	0.65 (0.64-0.67)
BAI	Oak - Hickory	Interior	0.5 (0.49-0.51)
BAI	Oak - Hickory	Anthropogenic Edge	0.7 (0.68-0.72)
BAI	Oak - Pine	All Edges	0.7 (0.65-0.76)
BAI	Oak - Pine	Interior	0.53 (0.49-0.57)
BAI	Oak - Pine	Anthropogenic Edge	0.77 (0.71-0.85)
BAI	Southern Conifers	All Edges	0.66 (0.59-0.74)
BAI	Southern Conifers	Interior	0.55 (0.5-0.62)
BAI	Southern Conifers	Anthropogenic Edge	0.67 (0.59-0.77)
BAI	Spruce - Fir	All Edges	0.44 (0.42-0.47)
BAI	Spruce - Fir	Interior	0.37 (0.35-0.4)
BAI	Spruce - Fir	Anthropogenic Edge	0.6 (0.54-0.68)
BA	All Forests	Interior	21.69 (21.35-22.05)
BA	All Forests	Edge	24.71 (24.3-25.13)
BA	All Forests	Anthropogenic Edge	26.93 (26.37-27.51)
BA	Aspen - Birch	Edge	16.86 (16.1-17.7)
BA	Aspen - Birch	Interior	16.02 (15.31-16.8)
BA	Aspen - Birch	Anthropogenic Edge	19.75 (18.41-21.31)
BA	Bottomland Forests	Edge	22.56 (21.49-23.74)
BA	Bottomland Forests	Interior	20.1 (19.18-21.11)
BA	Bottomland Forests	Anthropogenic Edge	23.14 (21.62-24.89)
BA	Northern Hardwood	Edge	26.95 (25.98-28)
BA	Northern Hardwood	Interior	23.27 (22.46-24.14)
BA	Northern Hardwood	Anthropogenic Edge	27.97 (26.74-29.32)
BA	Northern Pines - Hemlock	Edge	31.8 (30.04-33.77)
BA	Northern Pines - Hemlock	Interior	27.64 (26.14-29.34)
BA	Northern Pines - Hemlock	Anthropogenic Edge	34.29 (31.74-37.29)
BA	Oak - Hickory	Edge	25.69 (25.04-26.37)
BA	Oak - Hickory	Interior	22.08 (21.54-22.65)
BA	Oak - Hickory	Anthropogenic Edge	26.67 (25.89-27.5)

BA	Oak - Pine	Edge	26.69 (24.64-29.1)
BA	Oak - Pine	Interior	22.54 (20.83-24.55)
BA	Oak - Pine	Anthropogenic Edge	29.38 (26.67-32.71)
BA	Southern Conifers	Edge	25.65 (22.94-29.09)
BA	Southern Conifers	Interior	21.93 (19.75-24.65)
BA	Southern Conifers	Anthropogenic Edge	25.94 (22.58-30.48)
BA	Spruce - Fir	Edge	19.14 (17.97-20.47)
BA	Spruce - Fir	Interior	19.11 (17.96-20.41)
BA	Spruce - Fir	Anthropogenic Edge	22.35 (19.87-25.55)

Supplementary Table 4. Pre- and post-matching covariate distributions for all edges. Mean Edge and Mean Control show the average value of each predictor within Edge and Interior groups, respectively, before and after the matching process. Std. mean difference shows the difference in means.

**Continuous Variables**		**Mean Edge**	**Mean Control**	**SD Control**	**Std mean difference**	**eCDF Median**	**eCDF Mean**	**eCDF max**
**N. Deposition (kg ha-1)**	Before	9.08	8.15	2.58	0.37	0.12	0.10	0.13
	After	9.08	9.06	2.49	0.01	0.00	0.00	0.01
**Light**	Before	0.47	0.49	0.06	-0.28	0.03	0.05	0.12
	After	0.47	0.47	0.05	0.00	0.00	0.00	0.00
**Water**	Before	0.38	0.35	0.11	0.30	0.05	0.05	0.12
	After	0.38	0.38	0.10	0.00	0.00	0.00	0.00
**Temperature**	Before	0.68	0.70	0.09	-0.21	0.02	0.04	0.10
	After	0.68	0.68	0.09	0.00	0.00	0.00	0.00
**Forest Types**								
**Northern Hardwood**	Before	0.18	0.27	0.45	-0.23	0.04	0.04	0.09
	After	0.18	0.18	0.39	0.00	0.00	0.00	0.00
**Northern Pines - Hemlock**	Before	0.07	0.06	0.23	0.04	0.01	0.01	0.01
	After	0.07	0.07	0.25	0.00	0.00	0.00	0.00
**Oak - Hickory**	Before	0.39	0.33	0.47	0.12	0.03	0.03	0.06
	After	0.39	0.39	0.49	0.00	0.00	0.00	0.00
**Oak - Pine**	Before	0.04	0.03	0.18	0.01	0.00	0.00	0.00
	After	0.04	0.04	0.19	0.00	0.00	0.00	0.00
**Southern Pines - Other Conifers**	Before	0.02	0.02	0.13	0.04	0.00	0.00	0.01
	After	0.02	0.02	0.14	0.00	0.00	0.00	0.00
**Spruce - Fir**	Before	0.06	0.11	0.31	-0.21	0.02	0.02	0.05
	After	0.06	0.06	0.23	0.00	0.00	0.00	0.00

**Bottomland Forests**	Before	0.11	0.07	0.25	0.14	0.02	0.02	0.04
	After	0.11	0.11	0.31	0.00	0.00	0.00	0.00

Supplementary Table 5. Pre- and post-matching covariate distributions for anthropogenic edges. Mean Edge and Mean Control show the average value of each predictor within Edge and Interior groups, respectively, before and after the matching process. Std. mean difference shows the difference in means.

**Continuous Variables**		**Mean Edge**	**Mean Control**	**SD Control**	**Std mean difference**	**eCDF Median**	**eCDF Mean**	**eCDF max**
**N. Deposition (kg ha-1)**	Before	9.48	8.15	2.58	0.53	0.16	0.15	0.22
	After	9.48	9.47	2.50	0.01	0.00	0.00	0.01
**Light**	Before	0.47	0.49	0.06	-0.38	0.04	0.06	0.16
	After	0.47	0.47	0.05	0.00	0.00	0.00	0.00
**Water**	Before	0.37	0.35	0.11	0.20	0.03	0.03	0.10
	After	0.37	0.37	0.10	0.00	0.00	0.00	0.00
**Temperature**	Before	0.66	0.70	0.09	-0.49	0.04	0.08	0.22
	After	0.66	0.66	0.08	-0.01	0.00	0.00	0.00
**Forest Types**								
**Northern Hardwood**	Before	0.20	0.27	0.45	-0.19	0.04	0.04	0.07
	After	0.20	0.20	0.40	0.00	0.00	0.00	0.00
**Northern Pines - Hemlock**	Before	0.06	0.06	0.23	0.00	0.00	0.00	0.00
	After	0.06	0.06	0.24	0.00	0.00	0.00	0.00
**Oak - Hickory**	Before	0.47	0.33	0.47	0.28	0.07	0.07	0.14
	After	0.47	0.47	0.50	0.00	0.00	0.00	0.00
**Oak - Pine**	Before	0.04	0.03	0.18	0.02	0.00	0.00	0.00
	After	0.04	0.04	0.19	0.00	0.00	0.00	0.00
**Southern Pines - Other Conifers**	Before	0.02	0.02	0.13	0.04	0.00	0.00	0.01
	After	0.02	0.02	0.15	0.00	0.00	0.00	0.00
**Spruce - Fir**	Before	0.02	0.11	0.31	-0.54	0.04	0.04	0.08
	After	0.02	0.02	0.15	0.00	0.00	0.00	0.00
**Bottomland Forests**	Before	0.10	0.07	0.25	0.09	0.01	0.01	0.03
	After	0.10	0.10	0.29	0.00	0.00	0.00	0.00
