# Supplementary material for: Elevated growth and biomass along temperate forest edges
Source: Nat Commun. 2021 Dec 10;12:7181. doi: 10.1038/s41467-021-27373-7 (PMC8664805; doi:10.1038/s41467-021-27373-7)
Supplement: Supplementary file 3 — Reporting Summary [file 41467_2021_27373_MOESM3_ESM.pdf]

nature portfolio

Corresponding author(s): Luca L. Morreale

Last updated by author(s): 10/27/2021

# Reporting Summary

Nature Portfolio wishes to improve the reproducibility of the work that we publish. This form provides structure for consistency and transparency in reporting. For further information on Nature Portfolio policies, see our Editorial Policies and the Editorial Policy Checklist.

## Statistics

For all statistical analyses, confirm that the following items are present in the figure legend, table legend, main text, or Methods section.

| n/a | Confirmed | |
|---|---|---|
| ☐ | ☒ | The exact sample size (*n*) for each experimental group/condition, given as a discrete number and unit of measurement |
| ☐ | ☒ | A statement on whether measurements were taken from distinct samples or whether the same sample was measured repeatedly |
| ☐ | ☒ | The statistical test(s) used AND whether they are one- or two-sided<br>*Only common tests should be described solely by name; describe more complex techniques in the Methods section.* |
| ☐ | ☒ | A description of all covariates tested |
| ☐ | ☒ | A description of any assumptions or corrections, such as tests of normality and adjustment for multiple comparisons |
| ☐ | ☒ | A full description of the statistical parameters including central tendency (e.g. means) or other basic estimates (e.g. regression coefficient) AND variation (e.g. standard deviation) or associated estimates of uncertainty (e.g. confidence intervals) |
| ☐ | ☒ | For null hypothesis testing, the test statistic (e.g. *F*, *t*, *r*) with confidence intervals, effect sizes, degrees of freedom and *P* value noted<br>*Give P values as exact values whenever suitable.* |
| ☒ | ☐ | For Bayesian analysis, information on the choice of priors and Markov chain Monte Carlo settings |
| ☒ | ☐ | For hierarchical and complex designs, identification of the appropriate level for tests and full reporting of outcomes |
| ☒ | ☐ | Estimates of effect sizes (e.g. Cohen's *d*, Pearson's *r*), indicating how they were calculated |

*Our web collection on statistics for biologists contains articles on many of the points above.*

## Software and code

Policy information about availability of computer code

| | |
|---|---|
| Data collection | No software was used for data collection. |
| Data analysis | Statistical analyses and FIA data-processing were conducted in the R programming environment, version 3.4. Generalized linear model regressions were performed using the R stats package, version 3.4.3. Marginal effect estimates were calculated using R package ggeffects, version 1.1.1. Other GIS analyses were performed in the ESRI software ArcGIS Pro, version 2.4. The global analysis of forest fragmentation was performed in Google Earth Engine. The code used to analyze and process FIA data are not available publicly due to data privacy laws. |

For manuscripts utilizing custom algorithms or software that are central to the research but not yet described in published literature, software must be made available to editors and reviewers. We strongly encourage code deposition in a community repository (e.g. GitHub). See the Nature Portfolio guidelines for submitting code & software for further information.

## Data

Policy information about availability of data

All manuscripts must include a data availability statement. This statement should provide the following information, where applicable:

- Accession codes, unique identifiers, or web links for publicly available datasets
- A description of any restrictions on data availability
- For clinical datasets or third party data, please ensure that the statement adheres to our policy

The processed, post-matching FIA data generated in this study and used to generate Figures 1 and 2 have been deposited in the Harvard Forest Data Archive under accession code HF419 (url: https://harvardforest1.fas.harvard.edu/exist/apps/datasets/showData.html?id=HF419). The spatially-aggregated estimates of BAI presented in Figure 3c and summaries of global forest edge area displayed in Figure 4a are available in the HF Data Archive under accession code HF419. The un-fuzzed FIA location data are protected and are not available due to data privacy laws. Unprocessed FIA inventory data is available at https://apps.fs.usda.gov/fia/

datamart/. The National Land Cover Database land cover layer is at https://www.mrlc.gov/data. The forest cover map used for the global analysis is available on Google Earth Engine.

# Field-specific reporting

Please select the one below that is the best fit for your research. If you are not sure, read the appropriate sections before making your selection.

- [ ] Life sciences
- [ ] Behavioural & social sciences
- [x] Ecological, evolutionary & environmental sciences

For a reference copy of the document with all sections, see nature.com/documents/nr-reporting-summary-flat.pdf

# Ecological, evolutionary & environmental sciences study design

All studies must disclose on these points even when the disclosure is negative.

| | |
|---|---|
| Study description | We compare differences in tree biomass and growth between forest plots on the forest edge with those located in the forest interior. We use existing forest inventory data and create a quasi-experimental design, by matching treated plots (forest edges) with the same number of control plots (forest interior). We compare 6607 forest edge subplots with a subsample of forest interior plots (6607) from a population of 174,986 subplots, approximating a factorial design. We assess differences between these groups using a generalized linear model with a Gamma distribution. We then use the outputs of these models to extrapolate our results across our study region. |
| Research sample | The study uses forest inventory data collected by the US Forest Service (USFS). The Forest Inventory & Analysis (FIA) is a national forest inventory designed to be a statistically representative sample of the forest population of the United States. Our research sample includes all forested inventory plots within the Eastern Region of the USFS. Our rationale for this sample is that each region has its own remeasurement frequency and some differences in data collection protocols. Additionally, the data sharing agreement between Harvard University and the USFS for the true location of the FIA plots is constrained to this region. |
| Sampling strategy | We use all FIA subplots identifiable as located on the forest edge as our sample population to represent forest edges. We compare this to a subsample of interior forest plots, selected using a quasi-experimental causal inference framework (described in detail in the Methods document). The FIA is purposefully designed to be a representative sample of US forests. Therefore, as a proxy for predetermination of sample size, we leverage the statistically robust FIA design and the large quantity of independent samples (forest plots). |
| Data collection | Forest inventory data is collected by field crews hired or contracted by the USFS. Field crews measure all adult trees within fixed radius plots. Further details on FIA data collection are available on the USFS website (https://www.fia.fs.fed.us/) |
| Timing and spatial scale | In the USFS Eastern Region, forest plots were established beginning in the year 2000 and remeasured every 5-7 years, on average. For this analysis we use remeasurement data collected between 2010 through 2020. Individual tree measurements are taken within each plot, and we use data across the 20 states in the northeastern US that make up the USFS Eastern Region. This time period was bounded based on the most recently available FIA measurements, with 10 years prior to the most recent measurement selected as a start date to ensure the inclusion of a full remeasurement cycle for each plot. |
| Data exclusions | We exclude FIA subplots that are not identifiable as either directly adjacent to a forest edge or as located within the forest interior. We prevent pseudo-replication of subplots by ensuring the use of only one sub-plot per plot in our matching and subsequent analyses. Interior forest subplots are selected within the causal inference framework based on similarity to edge plots across observable confounding variables. Interior forest plots that are not selected for comparison are excluded from the analysis to prevent numerically biased comparisons between an oversampled control group with a smaller treatment sample. Finally, we exclude edge subplots that are under an area threshold of 30 square meters as determined by a sensitivity test. Exclusion criteria were not pre-established. |
| Reproducibility | To ensure reproducibility, all of our analyses are performed using programming that is documented and sharable (within the constraints of data privacy outlined in other sections). The code behind the analyses was re-run multiple times to ensure reproducibility. All recalculations of the analyses reproduced our reported findings. |
| Randomization | As detailed by FIA protocols, forest plot locations are selected randomly within a hexagonal grid across the United States. For our study, plots were allocated into two groups, edge and interior based on information within the FIA database identifying whether or not they were adjacent to a forest edge. To control for possible confounding covariates, we created a quasi-experimental design by subsampling the control (interior) group based on the covariates, using statistical matching to pair with the treatment group (edge plots). This ensures that our comparisons between the two groups are comparable along the dependent variables of interest (biomass and growth). |
| Blinding | To prevent bias in plot selection, locations of FIA forest plots are selected randomly by the USFS. Locations are not publicly available to prevent site interference that could bias data collection. |

Did the study involve field work? [ ] Yes [x] No

# Reporting for specific materials, systems and methods

We require information from authors about some types of materials, experimental systems and methods used in many studies. Here, indicate whether each material, system or method listed is relevant to your study. If you are not sure if a list item applies to your research, read the appropriate section before selecting a response.

## Materials & experimental systems

| n/a | Involved in the study |
|---|---|
| ☒ | ☐ Antibodies |
| ☒ | ☐ Eukaryotic cell lines |
| ☒ | ☐ Palaeontology and archaeology |
| ☒ | ☐ Animals and other organisms |
| ☒ | ☐ Human research participants |
| ☒ | ☐ Clinical data |
| ☒ | ☐ Dual use research of concern |

## Methods

| n/a | Involved in the study |
|---|---|
| ☒ | ☐ ChIP-seq |
| ☒ | ☐ Flow cytometry |
| ☒ | ☐ MRI-based neuroimaging |
